# Stretchable hermetic sealing for long-term soft-electronics protection

**DOI:** 10.1093/nsr/nwaf576

**Published:** 2025-12-23

**Authors:** Zhiming Zhang, Sihong Wang

**Affiliations:** Pritzker School of Molecular Engineering, The University of Chicago, USA; Pritzker School of Molecular Engineering, The University of Chicago, USA; Nanoscience and Technology Division, Argonne National Laboratory, USA; CZ Biohub, USA

Stretchable electronics represent a key frontier for next-generation technologies spanning applications from health monitoring to human–machine interfaces [1,2]. Reliable elastic encapsulation that prevents moisture and oxygen penetration is essential for ensuring the long-term operational stability of such devices [3]. Unfortunately, conventional elastomeric encapsulants inherently exhibit permeability of oxygen, moisture and other small molecules, as well as weak interfacial adhesion, resulting in poor barrier performance and mechanical delamination. Achieving metal-like hermeticity in a fully stretchable format has therefore remained a challenge.

In recent research published in *Nature Materials*, Xia and co-workers proposed a viscoplastic surface engineering strategy that enables aluminum-foil-level hermeticity in fully stretchable seals [4]. This strategy marks a departure from existing efforts to create stretchable hemostatic seals, which primarily relied on wrinkled stiff films and superhydrophobic fluorinated rubber sealants [5,6].

**Figure 1.**
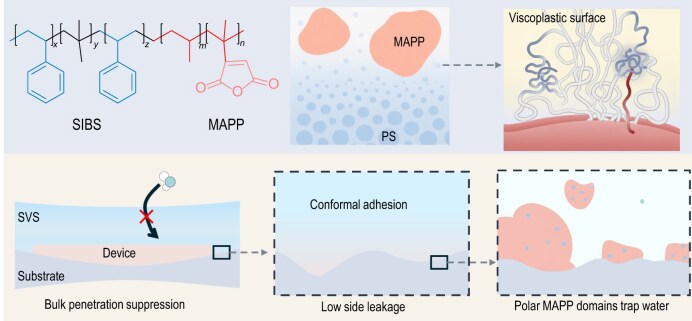
SVS provides hermetic and stretchable sealing via a viscoplastic surface created by controlled near-surface phase separation of polar plastics. PS: polystyrene.

The authors designed a surface-viscoplastic elastomer (SVS) by introducing maleic-anhydride-grafted polypropylene (MAPP) into poly(styrene–isobutylene–styrene) (SIBS) and controlling its phase separation to form discrete polar domains near the surface (Fig. 1). The viscoplastic surface of SVS enables strong and universal adhesion by allowing surface-chain flow to conform to counterpart topographies. Meanwhile, the subsurface MAPP-derived polar domains form reversible, energy-dissipative interactions with metals, plastics and elastomers, yielding defect-free and robust interfacial contacts. These polar domains also serve as localized traps for water molecules, strongly binding them as confined water clusters.

To further suppress residual moisture diffusion, the researchers incorporated molecular sieves and glycerol with ectoine into the SVS matrix to form a ‘scavenging SVS’ layer. These fillers not only adsorb trace amounts of water but also act as physical spacers between adjacent SVS films, thereby interrupting direct contact and eliminating continuous diffusion pathways. Owing to this synergistic scavenging and barrier effect, the scavenging SVS achieves an ultralow water-vapor transmission rate of below 10^−4^ g m^−2^ day^−1^, which remains unchanged after 50 000

stretching cycles at 100% strain or after 385 days in ambient air and 30 days underwater, demonstrating its robust long-term stability.

When applied to device encapsulation, the SVS seals provided long-term protection for a wide range of stretchable systems. Perovskite quantum dots retained their bright emission after more than 180 days of water immersion, hydrogel-based ionic thermoelectric generators operated stably for over a month in ambient air, and soft bioelectronic implants maintained functionality for 30 days *in vivo* without degradation. These demonstrations highlight the outstanding environmental stability enabled by the viscoplastic surface effect, ensuring reliable performance of stretchable electronics under prolonged exposure.

This work establishes a novel design principle for reconciling softness with hermeticity in stretchable materials. By engineering a dynamic viscoplastic surface enriched with polar domains, the authors achieved defect-free adhesion, suppressed molecular diffusion and unprecedented barrier performance in an elastomeric system. This strategy offers a universal approach to robust and durable encapsulation for wearable, biointegrated and soft electronic technologies. Moving forward toward fully solving the challenge of soft electronics, more effort on stretchable encapsulation materials is needed to maintain hermeticity under complex deformation modes and long-term environmental exposure.
